# Pharmacokinetics-adapted Busulfan-based myeloablative conditioning before unrelated umbilical cord blood transplantation for myeloid malignancies in children

**DOI:** 10.1371/journal.pone.0193862

**Published:** 2018-04-02

**Authors:** Joy Benadiba, Marc Ansari, Maja Krajinovic, Marie-France Vachon, Michel Duval, Pierre Teira, Sonia Cellot, Henrique Bittencourt

**Affiliations:** 1 Department of Hemato-Oncology Pediatric, Nice University Hospital, Nice, France; 2 Department of Pediatrics, Geneva University Hospital, Geneva, Switzerland; 3 Cansearch Research Laboratory, Geneva University Medical School, Geneva, Switzerland; 4 Hematology-Oncology Division - Centre de Cancérologie Charles-Bruneau, Ste-Justine Hospital, Montréal, Canada; 5 Clinical Pharmacology Unit, Ste-Justine Hospital, Montreal, Canada; 6 Department of Pediatrics, Faculty of Medicine - University of Montreal, Montreal, Canada; EFS, FRANCE

## Abstract

Unrelated umbilical cord blood transplantation (UCBT) is an alternative to provide transplants in children with acute leukemia or myelodysplastic syndrome who lack a related donor. Intravenous Busulfan (Bu) combined with therapeutic drug monitoring-guided dosing has been increasingly used, with more predictable bioavailability and better outcomes comparing to oral Bu. There is still an important variation in Bu pharmacokinetic between patients that is associated with an increased risk of toxicity and graft failure. The objective of the study was to analyze the impact of first-dose pharmacokinetic adapted myeloablative conditioning regimen of intravenous Bu on the different outcomes after transplantation. Data of 36 children who underwent allogeneic HSCT with Bu plus a second alkylating agent at Sainte Justine Hospital in Montreal, Canada, between December 2000 and April 2012 were analyzed. For children with high risk myeloid malignancies receiving an UCBT, first dose Bu pharmacokinetic seems to be a significant prognostic factor, influencing neutrophil (100% vs 67.9%) and platelet recovery (95.5% vs 70.5%), non-relapse mortality (0% vs 18.6%), EFS (64% vs 28.6%) and OS (81.3% vs 37.5%) for a first-dose steady-state concentration (Css) <600ng/mL vs >600ng/mL, respectively. These data reinforce the importance of Busulfan therapeutic drug monitoring-guided dosing in pediatric HSCT patients, particularly in the context of UCBT.

## Introduction

Unrelated umbilical cord blood transplant (UCBT) after a myeloablative conditioning regimen is a valid option for treatment of high-risk myeloid malignancies in children without a related donor, providing survival rates similar to unrelated HSCT donors[1–3].

Busulfan (Bu), a bi-functional alkylating agent, is used since 1980s in hematopoietic stem cell transplantation (HSCT) as part of the myeloablative conditioning regimen in acute myeloid leukemia (AML) and myelodysplastic syndromes (MDS), both in children and adults[4]. Bu-based conditioning regimens have been proposed in children as an alternative to total body irradiation (TBI), in order to avoid growth delay and other long lasting side effects related to the use of TBI [5, 6]. Intravenous (IV) Bu has replaced oral Bu in many HSCT centers since it has a more predictable bioavailability. IV Bu has been associated with higher event free survival (EFS), lower transplant-related mortality (TRM) and toxicity compared to oral Bu[7, 8]. However, there are still important variations in Bu pharmacokinetics (PK) between patients that are associated with increased risk of graft failure and toxicity, such as hepatic veno-occlusive disease (VOD) or graft versus host disease (GvHD)[9–11]. Furthermore, intra- and interpatient Bu PK variability seems to be more important in children[12, 13]. It has been hypothesized that variability in Bu PK and treatment outcomes might be predicted by genetic variants of enzymes involved in the metabolism of Bu[14]. To optimize treatment with Bu, many transplant centers use therapeutic drug monitoring (TDM) and subsequent dose adjustment.

The objective of this study was to analyze results of myeloablative conditioning regimen based on IV Bu for AML and MDS, after unrelated UCB transplantation in children, and to evaluate the impact of Bu PK on the different outcomes after HSCT.

## Patients and methods

This was a single-center study including 36 out of 49 children with a myeloid malignancy who underwent allogeneic HSCT with IV Bu as part of a myeloablative conditioning regimen at CHU Sainte-Justine in Montreal, Canada, between December 2000 and April 2012. From 2008, data were prospectively collected (Clinicaltrials.gov identifier: NCT01257854). Parents signed informed consent for collection and inclusion of their child’s HSCT data in our HSCT data registry. Approval from the Institutional Ethics Board was obtained to perform this study. Thirteen out of 49 patients who didn’t have signed informed consent were excluded from this study.

Myeloablative conditioning regimen consisted of 16 doses of IV Bu, every 6 hours, from day -9 to day -6 before HSCT, according to age (16mg/m^2^/dose in infants less than 3 months old, 0.8 mg/kg/dose if less than 1 year of age, 1 mg/kg/dose for children more than one year old and 0.8 mg/kg/dose for children more than 4 years old) followed by a PK-guided dose adjustment (based on this first dose) performed at the fifth dose level[7]. Bu was infused over 2 hours. Blood samples were collected for PK immediately before and at 15, 30, 60, 120, 180, and 240 minutes after the first dose. Plasma Bu concentrations were determined using a modified high-performance liquid chromatography assay[15] and was usually available before the fifth dose. Based on the first dose PK parameters, further doses of Bu were adjusted from the fifth dose onward to achieve a steady-state concentration (Css) between 600–900 ng/mL. This was achieved by estimating maximum and minimum plasma concentrations of Bu at the ninth dose based on first-dose clearance in a noncomportmental model for continuous IV infusion. A factor was calculated for adjustment of these concentrations to have a mean plasma concentration of 600–900 ng/mL at the ninth dose and then doses were adjusted with consideration of the adjustment factor. Bu PK was not done on subsequent Bu doses after first dose for most patients.

Bu was combined with Cyclophosphamide (Cy) with a cumulative dose of 200 mg/kg over 4 days, Melphalan 135 mg/m^2^ or Cy 120 mg/kg and Etoposide 30 mg/kg. Cyclosporin A and steroids were used as graft-versus host disease (GvHD) prophylaxis and 35 patients received antithymocyte globulins (ATG). Granulocyte colony-stimulating factor was used until neutrophils reached > 5x10^9^/L for 2 days after UCBT for all patients. Prophylaxis for seizure was initiated 24 hours before Bu IV and continued for at least 24 hours after the end of Bu with lorazepam or midazolam for all but one patient, who received phenytoin. Fluconazole was administered as part of a supportive care regimen after the last dose of Bu and antiemetics were routinely administered throughout the conditioning regimen. Acyclovir was given as prophylaxis for herpes virus, and trimethoprim/sulfamethoxazole for pneumocystis jiroveci prophylaxis. Ursodeoxycholic acid was given to every patient as VOD prophylaxis.

Neutrophil recovery was defined as the first of 3 consecutive days of absolute neutrophil count ≥0.5 x 10^9^/L. Platelet recovery was defined as the first of 7 consecutive days of platelet counts ≥50 x 10^9^/L without transfusion. Primary graft failure or rejection was defined by persistent pancytopenia with no evidence of hematologic recovery of donor cells beyond 42 days after transplantation and secondary graft failure by a rapid decrease in neutrophil count and chimerism after successful engraftment. Acute GvHD (aGvHD) grading was based on the 1994 Consensus Conference on Acute GvHD Grading[16]. VOD was diagnosed according to Seattle criteria[17]. Non-relapse mortality (NRM) was defined as all causes of deaths after transplant not related to relapse. Time to relapse was calculated from the time between transplant and relapse. Event-free survival (EFS) was defined as the time from transplant until death, relapse, or graft failure, whichever occurred first. Overall survival (OS) was the time between transplantation and last contact or death of any cause.

For study variables, median value/range was reported for continuous variable and frequency/percentage for categorical variable. For statistical analysis, each continuous variable was dichotomized in 2 groups based on median value. A median Css of 576.5ng/mL was observed after the first Bu dose in the cohort, which did not differ by more than 5% from the lower limit of the targeted Css range. Hence, 600 ng/mL was chosen as the cut-off to dichotomize patients groups. Cumulative incidence of neutrophil and platelet recovery, grade 2–4 aGvHD, NRM, relapse, hemorrhagic cystitis (HC), VOD, and lung toxicity were calculated using the cumulative incidence (CI) estimator, with death as a competitive event (relapse for NRM). Probabilities of OS and EFS were calculated using the Kaplan–Meier method and log-rank test in univariate analysis. Multivariate analysis for OS and EFS was performed using Cox proportional hazards regression. Variables included in the multivariate analysis included Css, median infused nucleated cells per Kg and HLA compatibility (both known to influence UCBT outcomes after UCBT). Variables with a p<0.05 in univariate analysis were also included in the model. Two-sided p values were represented and p<0.05 was considered as statistically significant. Statistical analyses were performed using IBM SPSS Statistics 20 (IBM Corp, Armonk, NY) and Easy R software[18]. Approval from the Institutional Ethics Board (“Comité d’éthique de la recherche du CHU Sainte-Justine”) was obtained to perform this study.

## Results

Median follow-up was 29.8 (0.9–113.4) months. Median age at transplant was 5.9 (0.6–19.3) years. Of 36 patients, 21 (58.3%) were male. Median weight was 25 (6.9–85.7) kg. There were 23 AML and 13 MDS. Cytogenetics were normal for 10 patients, five had monosomy7, four deletion 5q, four trisomy 21 and three MLL rearrangements. For four patients, cytogenetics were associated with a good prognosis, including one case with t(8;21), two with inversion 16, and one patient with t(15;17). Ten and 13 patients with AML were transplanted in first remission (CR1) or second remission (CR2)/ advanced phase of disease (>CR2 or in relapse), respectively. Thirty-three patients received a single UCBT. Eight, 13 and 15 patients received a 6/6, 5/6 and 3-4/6 HLA-matched graft. Median infused nucleated cells (NC) and CD34+ cells were 5.5 (0.51–29.09) x 10^7^/kg and 2.2 (0.77–25.5) x 10^5^/kg of recipient body weight, respectively. Bu was combined with Cy for 33 patients, Melphalan for 2 patients and Cy/Etoposide for one patient.

Table 1 summarizes the patients and transplant characteristics.

**Table 1:** Patients demographic characteristics (n = 36).

Demographic characteristics	Patients N(%) or median (min-max)
Gender	
Female	15 (41.7)
Male	21 (58.3)
Ethnic background	
White Caucasian	33 (91.7)
Asiatic	1
American Indian	1
Moroccan	1
Diagnosis	
AML	23(63.9)
MDS	13(36.1)
Cytogenetic characteristics	
Normal	10
Monosomy 7	5
Deletion 5q	4
Trisomy 21	4
Rearrangement MLL	3
Others	9
Cytogenetic characteristics associated with good prognosis	4
t(8,21)	1
Inv 16	2
t(15,17)	1
Age at HSCT (years)	5.9(0.6–19.3)
Umbilical cord blood transplant	
Single	33(91.6)
Double	3
HLA compatibility	
6/6	8(22.2)
5/6	13(36.1)
3-4/6	15(41.6)
NC dose x 10^9^/Kg	5.52(0.51–29.09)
CD 34 dose x 10^9^/Kg	2.22(0.77–25.49)
Disease status at HSCT for AML	
CR1	10
CR2 or advanced phase of disease (≥CR2 or relapse)	13
Conditionning	
Bu+Cy	33(91.7)
Bu+Mel	2
Bu+Cy+VP16	1
Css ng/mL	576 (399–1153)

AML, acute myeloid leukemia, MDS, myelodysplastic syndrome, CR1, first complete remission, CR2, second complete remission, Bu, Busulfan, Cy, Cyclophosphamide, Mel, Melphalan, HSCT, hematopoietic stem cell transplant, NC, nuclear cells, Css, steady-state concentration

Median Css at the first dose was 576.5 (399–1153) ng/mL. Twenty-two patients had a first-dose Css below 600 ng/mL, and 14 above. For 9 patients, the initial prescribed dose of Bu was not changed (n = 5) or changed by less than 10% (n = 4). For 3 patients, initial prescribed dose of Bu was decreased, and for 24 patients the initial prescribed dose of Bu was increased (median increase 25%—range: 14%–56.2%). Median Bu clearance was 4.17 (2.06–5.87)mL/min/kg.

**Fig 1:**
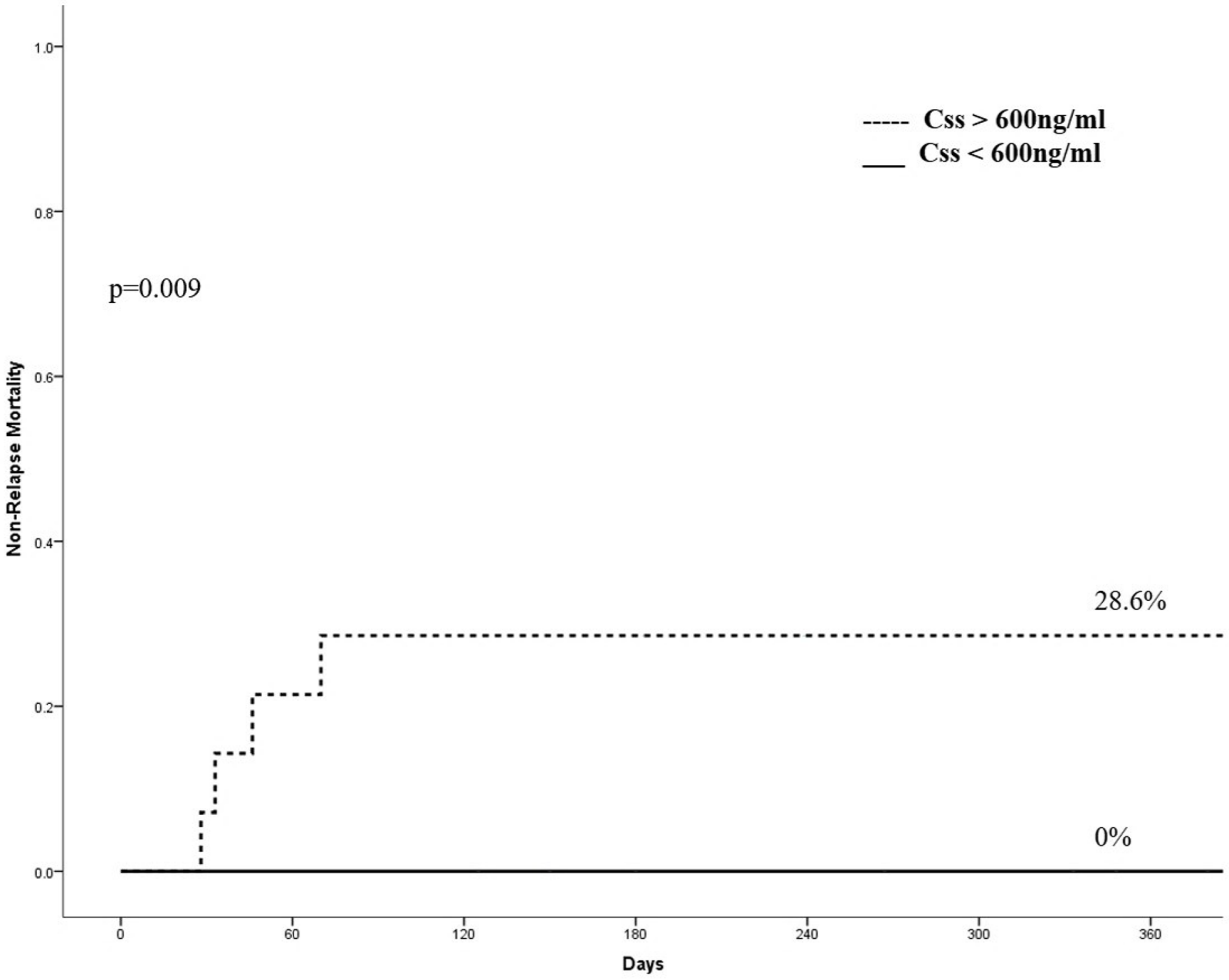
Non-relapse mortality according to concentration at the steady state.

Cumulative incidence (CI) of neutrophil recovery at the 60^th^ day post-transplant was 87.5% and platelet recovery at day +150 was 85.7%. Median time to neutrophil and platelet recovery was 22 days and 73 days, respectively. There were one primary and one secondary graft failure. CI of neutrophil recovery according to Css was 100% for Css <600 ng/mL, and 67.9% for Css > 600 ng/mL (p = 0.01) and CI of platelet recovery was 95.5% for Css < 600 ng/mL, and 70.5% for Css > 600 ng/mL (p = 0.04). No other variable was associated with neutrophil or platelet recoveries. Cumulative incidence of grade 2–4 aGvHD at day +180 was 15.1% and was not associated with any variable, or influenced by Bu PK. CI of hemorrhagic cystitis was 30.6%, and was significantly higher for patients with a Css > 600 ng/mL (50%) comparing to patients with Css < 600 ng/ml (18%—p = 0.04). Incidence of VOD was 6%, and occurred only in patients with Css > 600 ng/mL. Two cases of severe lung toxicity (acute respiratory distress syndrome) were observed, one patient on each group of Css. Cumulative incidence of relapse was 33.4% at 5 years and there was a trend of less relapse for patients with MDS (p = 0.08) and for those receiving a 6/6 compatible cord blood (p = 0.07). Cumulative incidence of NRM at 5 years was 11.1%. The only factor influencing NRM in univariate analysis, was Bu Css; the NRM was 0% vs. 28.6% for Css < and > 600 ng/mL, respectively (p = 0.009) (Fig 1).

**Fig 2:**
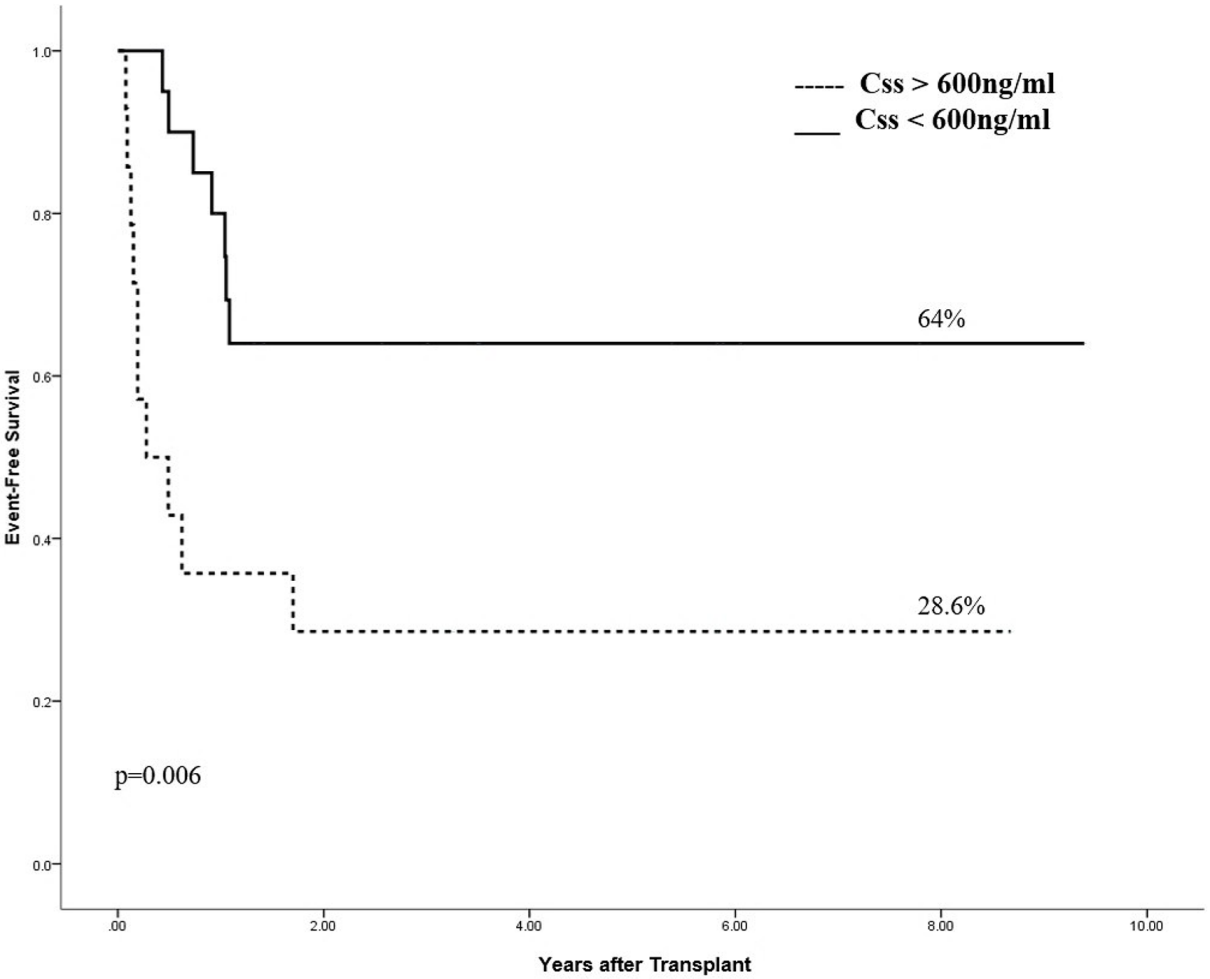
Event free survival according to concentration at the steady state.

**Fig 3:**
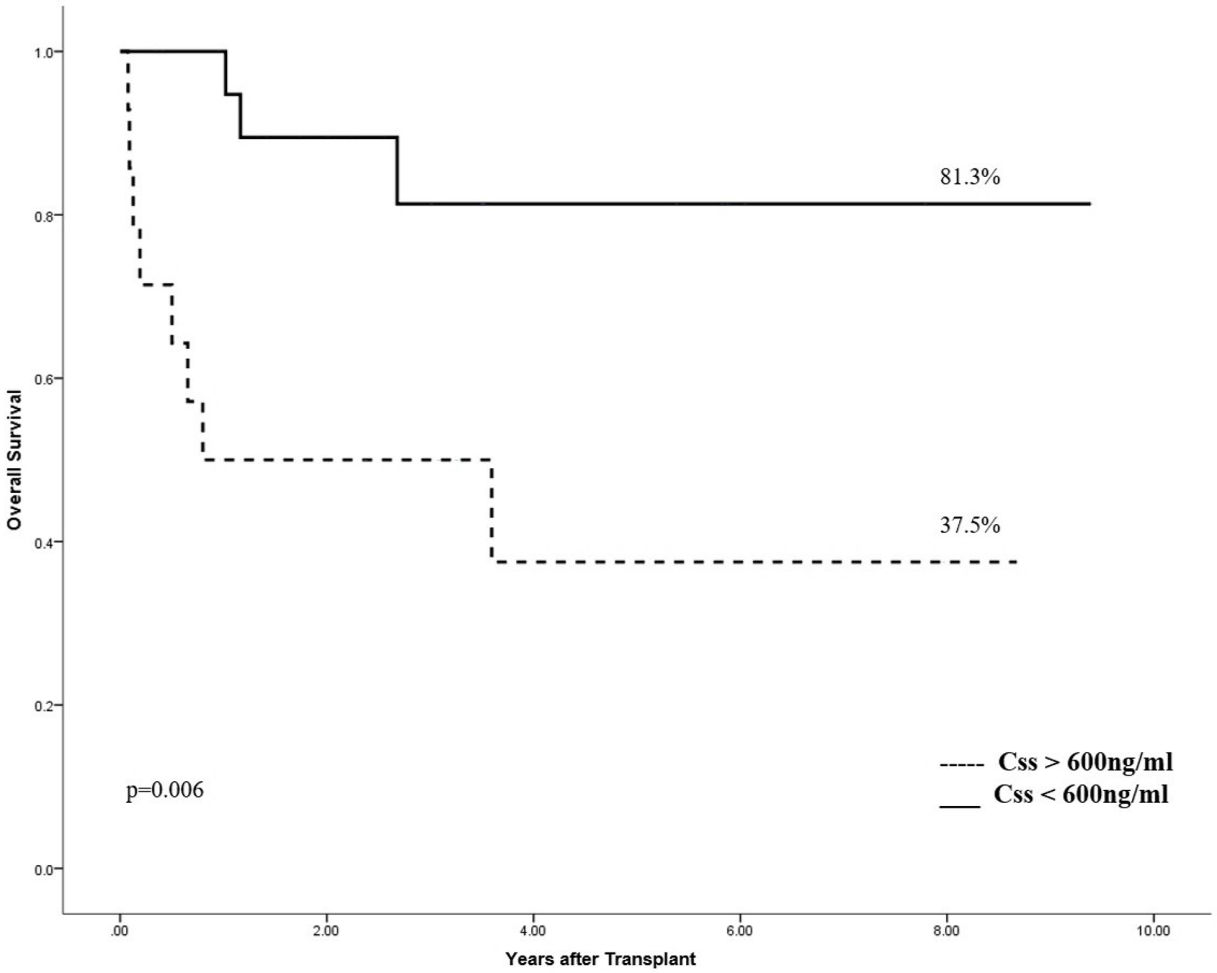
Overall survival according to concentration at the steady state.

Event-free survival (EFS) and overall survival (OS) were 50% and 63% respectively, for the whole population. Bu Css was shown to be the only predicting variable in univariate analysis: EFS: 64% vs. 29% for Css < and > 600 ng/mL, respectively (p = 0.006—Fig 2) and OS: 81% vs. 37.5% for Css < and > 600 ng/mL, respectively (p = 0.006- Fig 3).

In multivariate analysis, Css was the only variable to influence OS (HR: 5.2 [95% CI: 1.26–21.5] p = 0.02) and EFS (HR: 3.83 [95% CI: 1.33–11.05] p = 0.01).

## Discussion

Conditioning regimens including total body irradiation (TBI) in combination with high dose chemotherapy have been used in patients with AML since the 1970s, and such regimens are known to be effective in achieving engraftment and eradicating disease[6]. Bu-based myeloablative regimens given before HSCT, are used in children as a TBI alternative to avoid late side effects such as growth impairments, cataracts, endocrinopathies, cognitive delay, and increased incidence of secondary malignancies associated with exposure to irradiation[6, 19]. There is no evidence of an advantage of using TBI on children with AML beyond CR1, since no significant difference in outcomes (EFS, OS, relapse) comparing TBI with Busulfan based conditioning regimen has been reported[6].

Oral Bu has a large inter-individual variability, with higher plasma concentrations associated with toxicity, whereas lower concentrations result in an increased risk of graft failure or relapse[8, 12, 20–24]. This might be particularly important in UCBT that carries a higher risk of graft failure. Intravenous formulation of Bu has gained popularity as pharmacokinetics is more predictable[19]. Nevertheless, inter- and intra-individual variability still exists, mostly in children. IV Bu dose with adjustment based on first-dose pharmacokinetics reduces the variability of Bu exposure among patients[25–27], improving engraftment, EFS and survival rates[20, 25].

Bu is primarily metabolized by liver glutathione S-transferase (GST) enzymes, predominantly by its variant GSTA1. This process depletes hepatocyte glutathione stores. Bu metabolism is influenced by GST enzyme activity, age, disease condition and co-medication[13]. Body surface area and body weight also contribute to PK variability[28]. Children appear to have a higher Bu clearance, resulting in a lower systemic exposure. The higher GST activity in children might explain the difference[12, 29]. GST polymorphisms influencing GST activity could partly contribute to the unexplained Bu variability[25, 30, 31]. A study of the European Blood and Marrow Transplant Group, confirmed that GST gene variants (GSTA1 and GSTM1) influence Bu PK and outcomes of HSCT in children[13].

In this study, we showed that Bu Css of the first dose seems to influence different outcomes after UCBT. A Bu Css below 600ng/mL associates with a better EFS, OS, NRM, and hematopoietic (neutrophil and platelet) recovery. On the other hand, there is no association between a lower first-dose Css and graft failure. However, since patients in this series had received a graft with a total dose of cells according to recommendation for a UCBT, this might have prevented a graft failure in most of patients. Unfortunately, there are missing data concerning the PK results on subsequent Bu doses in our study to confirm that the first dose Css, rather than the cumulative received Bu dose, explains the difference on the different outcomes. In a previous study including children who underwent HSCT with UCBT and UBMT for malignancies and non-neoplastic diseases, it was showed that Bu concentrations of the majority of children were in the targeted therapeutic range in subsequent doses[7]. The prognostic effect of the measured Bu Css was not due to an effect on relapse, since it was not affected by Css, similarly to what has been already described before[22, 32]. Rather, Bu Css at first dose seems to influence toxicity, as NRM occurred only in patients with a first-dose Css > 600 ng/mL. In adults, an association between higher Bu exposure and toxicity had been showed[11, 33]. But in pediatric patients, most authors reported absence of this association[34, 35]. Since all but two patients received Cy after IV Bu, glutathione (GSH) depletion in tissues, due to oxidative stress, might also have aggravated the tissue damage by Cy, initiating toxic events such as VOD, mucositis, hemorrhagic cystitis, lung toxicity, and others. Thus, the NRM seen with Css > 600 ng/mL could in fact be related to Cy toxicity after a GSH depletion by Bu, associate or not with a direct effect of Bu itself. It is known that Cy toxicity is highly dependent on GSH levels[36]. As some GST polymorphisms are associated with a lower Bu clearance, we can speculate that a higher Css is a surrogate marker for these polymorphisms[13]. Replacing Cy by other chemotherapy, such as fludarabine or tailored the first dose with an algorithm including demographics data as well as pharmacogenomics might improve the results of UCBT after Bu conditioning.

We showed that cumulative incidence of neutrophil and platelet recovery was higher when Bu Css was < 600 ng/mL. In contrast, in a study on 45 adults with chronic myeloid leukemia, there was no influence of Bu plasma levels on engraftment[21]. Similarly, Perkins et al reported that there was no correlation between first dose Bu AUC and bone marrow or lymphocyte chimerism studies around day 30 or day 100 after transplant with Bu and Fludarabine as conditioning before HSCT in adults[37]. The higher graft failure rate for those with first dose Bu Css >600 ng/mL can be explained by the higher NRM in the peri-engraftment period for these patients.

Differences in population and conditioning regimen might explain the differences between studies. Notwithstanding, our results are consistent with some studies conducted in children concerning the PK monitoring and individualization of Bu dosage regimen, where no graft rejection or graft failure were observed in pediatric bone marrow transplant recipients who received a lower total dose of Bu than those usually recommended[5]. Pediatric PK studies are however not uniform and large enough to define the relationship between Bu exposure and target therapeutic levels in children associated with optimal outcomes. Our study, including mainly Caucasian patients who received HSCT with only unrelated umbilical cord blood, with a uniformly PK targeted IV Bu and mostly together with Cy as myeloablative conditioning regimen, is unique to better define association between Bu exposure and outcomes of allogeneic UCBT. These are results from a single center, with uniform supportive care.

In conclusion, UCBT remains a useful treatment to patients with high-risk myeloid malignancies without a matching sibling donor, providing survival rates comparable to other graft sources. For patients receiving Bu and Cy as conditioning regimen before UCBT, initial first dose Bu PK (before adjustment to the cumulative dose) seems to be a significant prognostic factor in children, influencing neutrophil and platelet recovery, NRM, EFS and OS. A direct toxic effect of Bu and/or a synergistic toxic effect with Cy might explain the worst outcome when first dose IV Bu has a Css higher than 600ng/mL. It will be important to validate these results in a multicenter larger study and also to compare these results with patients who received a fixed dose of Bu. Finally, our data reinforces the importance of Bu therapeutic drug monitoring-guided dosing in pediatric HSCT patients.
